# The effects of acupoint catgut embedding therapy on anthropometric parameters and endocrine function in obese women: a systematic review and meta-analysis

**DOI:** 10.3389/fnut.2025.1583556

**Published:** 2025-07-01

**Authors:** Shuangqiu Li, Hongfei Zhou, Muhan Zhou, Liangliang Lou, Li Luo, Shan Ji

**Affiliations:** ^1^Liaoning University of Traditional Chinese Medicine, Shenyang, China; ^2^Liaoning Hospital of Traditional Chinese Medicine, Shenyang, China; ^3^Shenyang Second Traditional Chinese Medicine Hospital, Shenyang, China; ^4^Zhongshan Qu People's Hospital, Zhongshan, China; ^5^The Fifth People's Hospital of Shenyang, Shenyang, China

**Keywords:** acupoint embedding therapy, obesity, women, BMI, lipid profile, hormone, meta-analysis, systematic review

## Abstract

**Purpose:**

This study aimed to assess the effect of acupoint catgut embedding therapy (ACET) on anthropometric parameters and endocrine function in obese women through a systematic review and meta-analysis.

**Methods:**

A comprehensive search was conducted across international and Chinese databases [CNKI, Wanfang, Weipu, Sinomed, PubMed, Embase, Cochrane Library, Web of Science (WOS), and Scopus]. The search terms included “female,” “women,” “catgut implantation at acupoint,” “catgut embedding,” “acupoint embedding therapy,” “obesity,” “adiposity,” and “body weight,” etc. Studies included in this analysis were randomized controlled trials (RCTs) assessing the effects of ACET on obesity indicators such as body mass index (BMI), waist-to-hip ratio (WHR), lipid profiles such as triglycerides (TG), total cholesterol (TC), high-density lipoprotein (HDL), and low-density lipoprotein (LDL), and hormone levels such as luteinizing hormone (LH), follicle-stimulating hormone (FSH), testosterone (T), and estradiol (E2). We used ROB 2.0 to assess the risk of bias. Data was analyzed using weighted mean differences (WMD) and risk ratios (RR) to measure effect sizes, and heterogeneity was assessed using *I*^2^ statistics. Conduct sensitivity analysis, publication bias testing, and subgroup analysis on indicators with high heterogeneity to explore the sources of heterogeneity.

**Results:**

Twenty-three studies involving over 2,000 obese women were included. Risk of bias assessment revealed generally low bias in randomization and measurement domains, though selective reporting and missing data handling raised concerns in some studies. ACET significantly reduced BMI [−1.72 (95% CI: −2.13, −1.31)] and WHR [WMD −0.016 (95% CI: −0.034, 0.001)], with high heterogeneity in BMI analysis (*I*^2^ = 92.3%). Subgroup analyses suggested that heterogeneity decreased in different control groups and different treatment courses, such as diet guidance (*I*^2^ = 0.0%) and 12-week treatment duration (*I*^2^ = 32.9%). Publication bias assessments (Begg's and Egger's tests) indicated no significant bias for most indicators. However, the clinical efficacy rate showed potential publication bias upon trim-and-fill adjustment, though the effect remained significant. ACET significantly reduced TG and TC but not HDL, LDL, or insulin resistance. Hormonal changes included decreased LH and FSH and increased E2.

**Conclusion:**

Our meta-analysis demonstrates that ACET significantly improves anthropometric parameters and endocrine function in obese women, though it does not significantly impact lipid metabolism or insulin resistance. The therapy's influence on female hormones may contribute to its efficacy in obesity treatment, highlighting the need for further studies to explore long-term effects and mechanisms.

**Systematic review registration:**

PROSPERO, identifier: CRD42025640157.

## 1 Introduction

Obesity is a complex and multifactorial condition that continues to pose a significant public health challenge globally (1). Defined by a body mass index (BMI) of 30 kg/m^2^ or higher, obesity has seen alarming increases in prevalence over the past decades (2). As reported by the World Health Organization (WHO), obesity rates have tripled since 1975, with over 650 million adults classified as obese globally by 2021 (1, 3). This rise has directly contributed to the escalating burden of non-communicable diseases, including cardiovascular disease, type 2 diabetes, hypertension, and certain cancers, all of which are further straining healthcare systems worldwide (1, 3, 4). Particularly concerning is the disproportionate impact of obesity on women, as they are more susceptible to hormonal imbalances and metabolic disturbances that often accompany excess body fat (5). Women with obesity are at an increased risk for reproductive health problems, including polycystic ovary syndrome (PCOS), menstrual irregularities, and infertility (6). Furthermore, obesity is closely linked to mental health issues, such as depression and anxiety, which are frequently associated with body image concerns (7). Along with these concerns, obesity can disrupt metabolism, causing insulin resistance and unhealthy blood fat levels—both of which raise the risk of heart disease and type 2 diabetes (8).

Current strategies for managing obesity typically include lifestyle modifications, pharmacotherapy, and bariatric surgery (9). Lifestyle changes, such as dietary modifications and physical activity, remain the cornerstone of obesity treatment; however, these interventions often yield only modest and temporary results (10). Pharmacological therapies, including orlistat and GLP-1 receptor agonists, can assist in weight loss but usually come with side effects and limited long-term effectiveness (11). Bariatric surgery, such as gastric bypass and sleeve gastrectomy, offers more substantial and sustained weight loss but is invasive and carries surgical risks (12). Despite these existing treatments, maintaining weight loss remains a challenge for many, sparking greater interest in alternative therapies (13). Medications and surgeries may lead to fast results, but they frequently bring side effects ranging from stomach troubles to neurological complications.

Acupoint catgut embedding therapy (ACET) offers an alternative approach to managing obesity. Studies suggest that ACET, a specialized acupuncture technique, enhances insulin sensitivity, promotes satiety, and helps reduce food intake (14, 15). Recent randomized controlled trials (RCTs) have shown that patients with simple obesity who receive electroacupuncture combined with diet and exercise therapy experience greater weight loss compared to those who only receive diet and exercise (16). What's more, ACET appears to work particularly well for belly fat, with studies showing stronger results compared to other approaches (17). Another plus is that patients only need treatment every week or two, making it much easier to stick with than daily therapies, and this consistency likely boosts its effectiveness. Studies indicate ACET might help with several obesity-related problems women often face–like insulin resistance, cholesterol issues, and hormone fluctuations (18). That said, while early results look good, research findings have been mixed so far, and we still need more conclusive evidence. With studies showing mixed results, a meta-analysis gives us a way to thoroughly examine the research. By pooling data together, we can better identify trends, clarify whether ACET is truly effective against obesity, and evaluate how it impacts body composition and endocrine function.

## 2 Methods

This systematic review was conducted according to the PRISMA guidelines for systematic reviews and Meta-analyses (19). Additionally, it was registered with the international prospective register for reviews (PROSPERO), under registration number CRD42025640157.

### 2.1 Literature search strategy

We conducted a comprehensive literature search across Chinese and international databases to identify relevant studies for this meta-analysis. The search was carried out in eight databases: CNKI, Wanfang, Weipu (VIP), CBM, PubMed, Embase, Cochrane Library, Web of Science (WOS), and Scopus, with a cutoff date for publication up to October 1, 2024. The search strategy was designed to capture studies on the target population and intervention. For the population, the search terms included “female” and “women.” The intervention-related terms included “catgut implantation at acupoint,” “catgut embedding,” “catgut-implantation,” “catgut-point embedding,” and “acupoint embedding therapy.” In terms of outcomes, we used terms like “obesity,” “adiposity,” “body weight,” “abdominal obesity,” “central obesity,” and “visceral obesity.” The Complete Search Strategies were provided in Supplementary material 1.

### 2.2 Inclusion and exclusion criteria

Studies involving adult female participants aged between 18 and 60 years were included, diagnosed with obesity or overweight, defined by a BMI > 25 kg/m^2^. The intervention must involve acupoint catgut embedding therapy, which entails the implantation of absorbable surgical sutures (e.g., catgut or other absorbable threads) at specific acupoints. This may be combined with other acupuncture-related external therapies (such as needling, moxibustion, etc.). Only RCTs were included. Studies were required to report outcomes related to obesity or metabolic control. The primary outcome measures were BMI, waist-to-hip ratio (WHR), and clinical efficacy rate (the practical definition is the population with a weight loss of 3 kg or more). Secondary outcome measures included triglycerides (TG), total cholesterol (TC), high-density lipoprotein (HDL), low-density lipoprotein (LDL), homeostatic model assessment of insulin resistance (HOMA-IR), luteinizing hormone (LH), Testosterone (T), follicle-stimulating hormone (FSH), and estradiol (E2). Only studies published in English or Chinese were included to facilitate data extraction and analysis.

Exclusion criteria comprised studies focusing on non-female populations or participants under 18 years. Also excluded were studies that did not utilize acupoint embedding therapy or combined this with other treatments, such as pharmacological or surgical interventions. Non-randomized controlled trials, cohort studies, case-control studies, or those lacking a control group were excluded, as were studies with incomplete data or high risks of bias.

### 2.3 Data extraction and quality assessment

Data extraction was performed independently by two reviewers to ensure accuracy, with disagreements resolved by a third reviewer. We extracted study characteristics [author(s), year, country, study design, sample size, participant demographics, and intervention details], outcome measures (changes in BMI, waist-to-hip ratio, clinical efficacy rate, triglycerides, cholesterol, insulin resistance, and reproductive hormones), statistical data (means, standard deviations, effect sizes, and 95% confidence intervals), and adverse events. The quality of the studies was assessed using the Cochrane Risk of Bias 2.0 (ROB 2.0) tool, evaluating bias in five domains: (1) randomization, (2) intervention deviations, (3) missing outcome data, (4) outcome measurement, and (5) selective reporting. Each study had a low, high, or unclear risk of bias in each domain.

### 2.4 Statistical analysis methods

We used the weighted mean difference (WMD) for continuous outcomes and risk ratio (RR) for dichotomous outcomes as effect measures. The WMD was applied to compare changes in continuous variables such as BMI, WHR, and metabolic markers (e.g., triglycerides, cholesterol levels) between the intervention and control groups. For dichotomous outcomes, such as the clinical efficacy rate, the RR was used to compare the proportion of participants achieving significant improvements. A random-effects model was implemented when significant between-study heterogeneity was detected (*I*^2^ > 50%), while a fixed-effects model was adopted when heterogeneity was negligible. The heterogeneity of the studies was assessed using the *I*^2^ statistic, with values above 50% indicating substantial heterogeneity. If significant heterogeneity was observed, subgroup analyses based on factors such as study design, intervention duration, and participant characteristics were conducted to explore potential sources of variability. Additionally, sensitivity analyses were performed by excluding studies with a high risk of bias to assess their impact on the overall results. Publication bias was assessed using Egger's test and funnel plots. All statistical analyses were performed using STATA 15, and a *P*-value of < 0.05 was considered statistically significant.

## 3 Results

### 3.1 Characteristics of included studies

**Figure 1:**
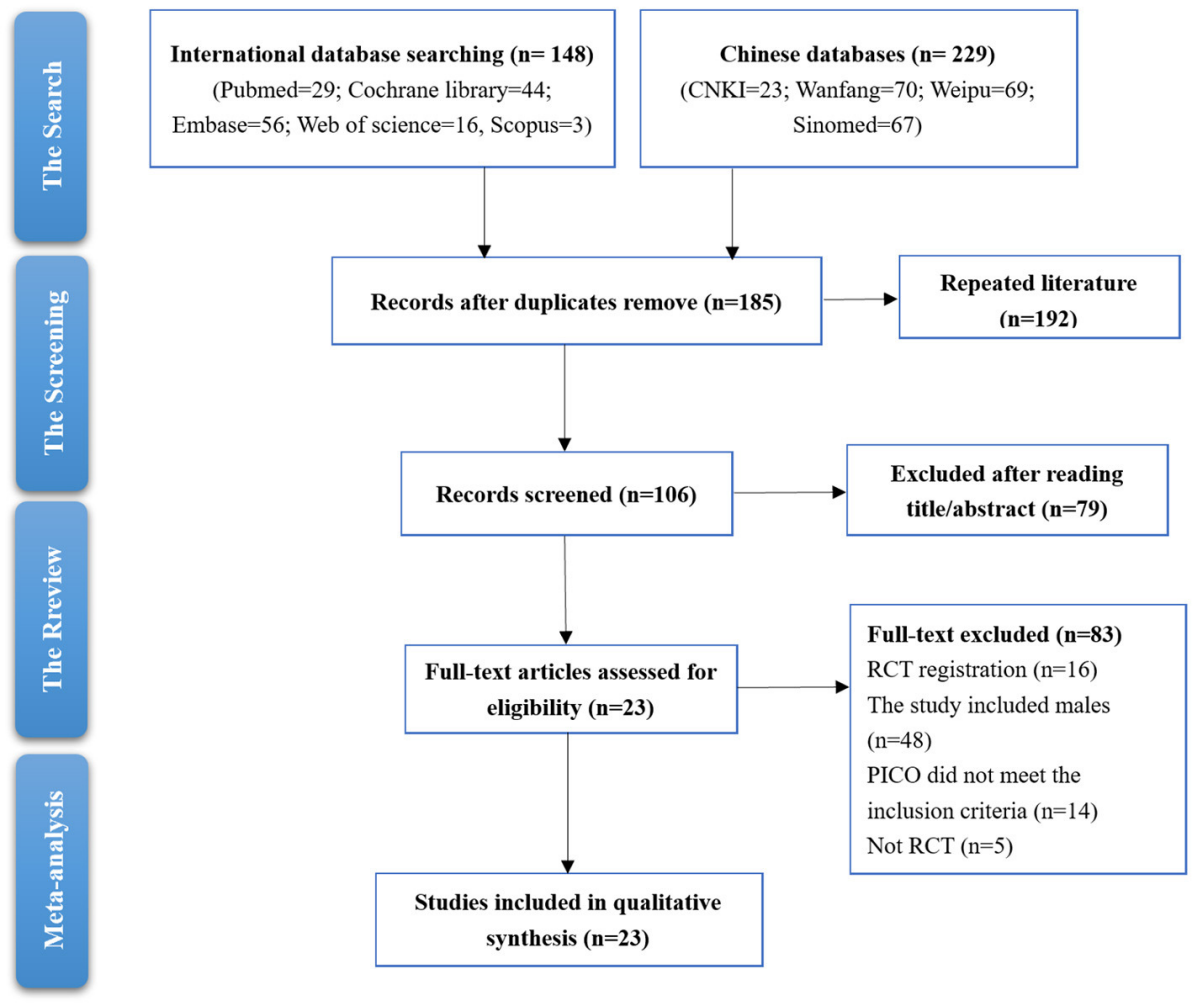
Studies screening process.

The study screening process involved a thorough examination of records from both international and Chinese databases. Initially, a total of 148 records were retrieved from international databases and 229 from Chinese databases. After removing duplicates, 185 records remained for further screening. A total of 106 records were screened after reading title and abstracts. Upon reviewing full text, 83 records were excluded. Eighty-three full-text articles were excluded for various reasons: 16 lacked RCT registration, 48 included males, 14 did not meet the PICO criteria, and five were not RCTs. Ultimately, 26 studies were included in the qualitative synthesis for the meta-analysis. The full-text articles of 23 studies were then assessed for eligibility (Figure 1).

The characteristics of included studies are as follows. The sample sizes ranged from 40 to 300 participants, with an age range typically from 25 to 52 years. The BMI values at baseline ranged from ~26 to 38 kg/m^2^. The predominant treatment was catgut embedding therapy, which was often combined with additional measures such as dietary guidance, moxibustion, or acupuncture. The type of suture material used in these interventions varied, with most studies employing absorbable catgut (sizes 2/0 or 4-0). The duration of treatment weeks, with the frequency of intervention typically ranging from once every week to once every 2 weeks. Some studies utilized additional therapies, such as on or acupuncture, either alone or in combination with catgut embedding. The control groups included sham catgut embedding, placebo acupuncture, and other basic treatments. The primary outcomes measured across studies included BMI, waist circumference, waist-to-hip ratio, metabolic indicators (lipid profiles such as total cholesterol, triglycerides, HDL, LDL, and fasting blood glucose), and hormone levels (such as estradiol, luteinizing hormone, and follicle-stimulating hormone; Table 1).

**Table 1:** Basic characteristics of included studies.

**Study**	**Characteristics of obese women**	**Intervention group**	**Control group**	**Outcome**
Chen 2018 (20)	90 cases. age from 39 to 43 years and had an average BMI of 30 kg/m^2^	Catgut embedding. Chromic catgut (UNIK surgical sutures MFG.CO.) size 0. 6 weeks	Sham catgut embedding. 6 weeks	BMI, WHR, TG, TC, HDL, LDL, AE
Chu 2014 (48)	50 cases. IC: 29 ± 5 years, 25.78 ± 4.81 kg/m^2^; CG: 27 ± 5 years, 26.88 ± 4.53 kg/m^2^	Catgut embedding. No. 0 medical catgut. 2 months	Electroacupuncture. 0.30 mm × 40 cm 75 mm filiform needle. 2 months	BMI, WHR, clinical efficacy
Deng 2021 (49)	60 cases. IG: 49.5 ± 2.5 years, 31.05 ± 1.15 kg/m^2^; CG: 50.2 ± 2.8 years, 31.08 ± 1.17 kg/m^2^	Catgut embedding+ Dietary guidance. Surgical suture (polyglycolic acid PGA suture 4-0). 3 months	Dietary guidance. 3 months	BMI, E_2_, LH, FSH
Du 2011 (50)	64 cases. IG: 50.3 ± 3.0 years, 30.61 ± 3.32 kg/m^2^; CG: 49.8 ± 3.2 years, 30.38 ± 2.97 kg/m^2^	Catgut embedding+ Dietary guidance. Medical catgut. 12 weeks	Dietary guidance. 12 weeks	BMI, clinical efficacy
Jessica 2014 (51)	99 cases. 35.9 ± 7.8 years. Mean BMI was between 33.4 ± 1.3 kg/m^2^ and 38.6 ± 2.6 kg/m^2^	Catgut embedding+ Moxibustion. Chromic catgut strand 00. 6 weeks	Sham acupuncture. 6 weeks	BMI, WHR, TC, HOMA-IR
Jin 2024 (23)	82 cases. IG: 26.3 (23.2, 27.4) kg/m^2^, CG: 25.5 (23.95, 27.58) kg/m^2^	Catgut embedding. Absorbable surgical suture (4–0 RA-1012). 8 weeks	The placebo embedding needle. 8 weeks	BMI, WHR, TG, TC, HDL, LDL, HOMA-IR
Qin 2016 (21)	41 cases. IG: 28.33 ± 5.12 years; 27.44 ± 2.35 kg/m^2^; CG: 28.77 ± 4.77 years, 27.06 ± 2.01 kg/m^2^	Catgut embedding 3-0, 3 months	Kidney-supplementing and blood-quickening decoction. 3 months	BMI, WHR, TG, TC, HDL, LDL, HOMA-IR, clinical efficacy, AE
Fu 2024 (52)	90 cases. IG: 31.41 ± 2.56 years; 26.35 ± 1.0 kg/m^2^; CG: 27.33 ± 2.41 years, 26.52 ± 1.12 kg/m^2^	Catgut embedding+ IVF-ET. Medical absorbable protein thread	IVF-ET	LH, T, FSH, TC, TG
Han 2022 (53)	68 cases. IG: 42.5 ± 10.3 years; CG: 39.2 ± 10.7 years	Catgut embedding. No. 2 absorbable surgical suture. 8 weeks	Sham catgut embedding. 8 weeks	BMI
Jin 2023 (54)	90 cases. IG: 27.58 ± 2.46 years; CG: 27.62 ± 2.47 years	Catgut embedding+ ACU. Medical “4-0” absorbable surgical suture+ 1.5~ 2.0 inch filiform needle. 8 weeks	Catgut embedding. Medical “4-0” absorbable surgical suture. 8 weeks	Clinical efficacy, T, LH, FSH, AE
Lai 2015 (55)	40 cases. IG: 37.34 years; 28.21 ± 2.84 kg/m^2^; CG: 36.13 years, 28.31 ± 2.73 kg/m^2^	Catgut embedding+ ACU. “4-0” sterile absorbable catgut + 0.3 × 40 mm disposable acupuncture needle. 8 weeks	Catgut embedding. “4-0” sterile absorbable catgut. 8 weeks	BMI, clinical efficacy
Liu 2013 (56)	60 cases. 27.15 years; IG: 29.3 ± 3.7 kg/m^2^; CG: 29.2 ± 3.2 kg/m^2^	Catgut embedding. The No. 2-0 medical catgut. 3 months	Electroacupuncture. 3 months	BMI, WHR, clinical efficacy
Liu 2023 (57)	60 cases. IG: 26.8 ± 4.01 years; 27.58 ± 30.31 kg/m^2^; CG: 27.45 ± 4.02 years, 27.96(26.79, 30.12) kg/m^2^	Catgut embedding. Line 4-0. 3 months	Needle knife. 3 months	BMI, WHR, clinical efficacy
Lu 2020 (58)	106 cases. IG: 52.0 ± 4.1 years; 30.17 ± 2.24 kg/m^2^; CG: 51.2 ± 4.6 years, 30.21 ± 2.19 kg/m^2^	Catgut embedding+ Basic treatment. Absorbable gut No. 4-0. 12 weeks	Basic treatment. Mirtazapine tablet+ Nilestriol tablets. 12 weeks	BMI, E_2_, FSH, LH
Song 2018 (22)	300 cases. IG: 33.2 ± 2.6 years; 28.8 ± 2.9 kg/m^2^; CG: 33.5 ± 2.5 years, 28.7 ± 2.5 kg/m^2^	Catgut embedding+ Massage. Absorbable medical catgut (size 4-0). 8 weeks	Massage. 8 weeks	BMI, WHR, TG, TC, clinical efficacy, AE
Wang 2022 (39)	98 cases. IG: 52.61 ± 3.06 years; 30.66 ± 1.59 kg/m^2^; CG: 52.64 ± 3.02 years, 30.72 ± 1.58 kg/m^2^	Catgut embedding+ Basic treatment. Absorbable medical catgut (Specification: 2/0). 12 weeks	Basic treatment. Targeted exercise and diet adjustment. 12 weeks	BMI, WHR, TG, TC, LDL, clinical efficacy
Wang 2014 (40)	240 cases. IG: 36.7 ± 10.1 years; 33.21 ± 3.17 kg/m^2^; CG: 37.7 ± 9.2 years, 32.74 ± 2.65 kg/m^2^	Catgut embedding+ Aerobic exercise. Catgut. 3 months	Aerobic exercise. 3 months	Clinical efficacy
Wang 2013 (41)	90 cases. IG: 39.1 ± 11.8 years; 28.226 ± 2.63 kg/m^2^; CG: 37.9 ± 11.5 years, 28.226 ± 2.63 kg/m^2^	Catgut embedding +Cupping. No. 0-1 medical catgut. 3 months	Acupuncture. 3 months	BMI, clinical efficacy
Xu 2021 (42)	70 cases. IG: 42.48 ± 2.69 years; 24.25 ± 4.62 kg/m^2^; CG: 42.51 ± 2.64 years, 25.42 ± 4.58 kg/m^2^	Catgut embedding. Protein molecular line	Electroacupuncture	BMI, WHR
Xue 2017 (43)	57 cases. IG: 34 ± 9.61 years; 27.23 ± 2.71 kg/m^2^; CG: 38 ± 12.48 years, 26.43 ± 2.35 kg/m^2^	Catgut embedding + Cupping. Absorbable surgical thread for hand surgery+ No. 2 cupping. 3 months	Catgut embedding. Cupping. Absorbable surgical thread for hand surgery. 3 months	BMI, clinical efficacy
Yang 2023 (44)	134 cases. IG: 29.85 ± 5.64 years; 29.41 ± 2.81 kg/m^2^; CG: 30.31 ± 5.94 years, 29.57 ± 3.09 kg/m^2^	Catgut embedding+ Embedding beans. The Complete shell-free vaccinium seeds. 3 months	Catgut embedding. 3 months	HOMA-IR
Yu 2017 (45)	140 cases. IG: 51.07 ± 4.96 years; 30.38 ± 3.21 kg/m^2^; CG: 50.38 ± 5.42 years, 30.21 ± 3.34 kg/m^2^	Catgut embedding+ Basic treatment. 2/0 absorbable catgut (manufactured by B. Braun Melsungen AG). 2/0 absorbable catgut (manufactured by B. Braun Melsungen AG). 12 weeks	Basic treatment. Exercise therapy+ Diet control+ Mirtazapine tablets (Huayu Pharmaceutical Co., Ltd., National Medicine Approval No. H20041656, specification 30 mg)+ Nilestriol tablets (Beijing Sihuan Pharmaceutical Co., Ltd., National Medicine Approval No. H11020124, specification 2 mg). 6 months	BMI, WHR, E_2_, LH, FSH
Zhang 2017 (46)	71 cases. IG: 34 ± 6 years; 27.73 ± 1.52 kg/m^2^; CG: 32 ± 5 years, 22.43 ± 1.56 kg/m^2^	Catgut embedding+ Basic treatment. 4-0 suture thread (lot number: thread VCP422). 20 weeks	Basic treatment. Routine postpartum nutrition counseling guidance. 20 weeks	BMI, WHR

BMI, body mass index; WHR, waist-to-hip ratio; TG, triglycerides; TC, triglycerides; HDL, high-density lipoprotein; LDL, low-density lipoprotein; HOMA-IR, homeostasis model assessment of insulin resistance; LH, luteinizing hormone; FSH, follicle-stimulating hormone; T, testosterone; E_2_, estradiol; AE, adverse events.

### 3.2 Bias of risk

**Figure 2:**
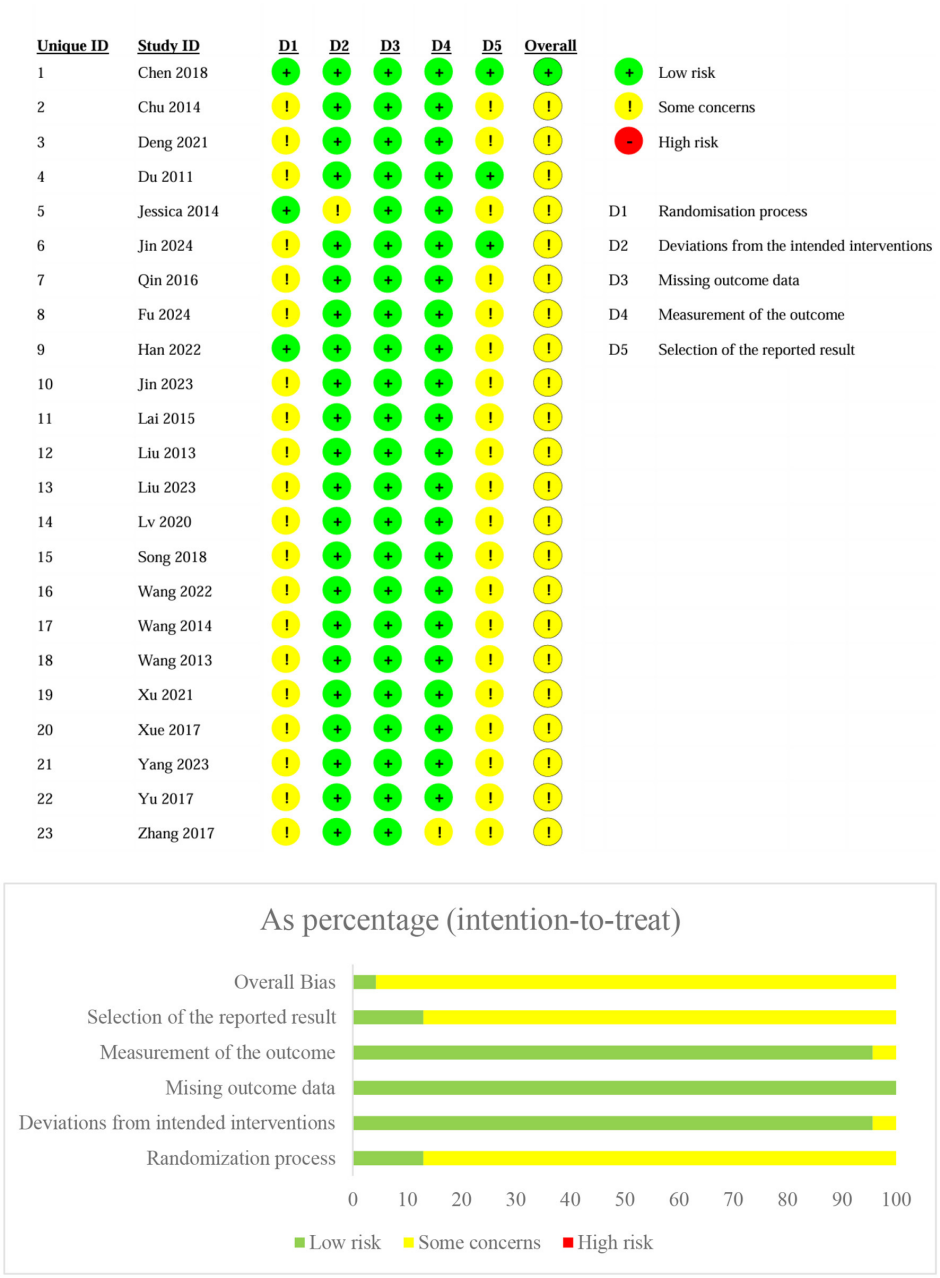
Bias of risk assessment.

The majority of studies showed low risk of bias in most domains, particularly for the randomization process, where most studies adhered to appropriate randomization methods, ensuring unbiased selection of participants. Additionally, most studies employed reliable and valid measurement methods. The missing data were not adequately addressed or imputed, which may have impacted the robustness of the findings. Finally, the “Selection of the reported result” domain showed mixed results, with some studies exhibiting a high risk of bias due to selective reporting of outcomes. In these cases, certain results may have been emphasized over others, potentially skewing the overall findings of the meta-analysis (Figure 2).

### 3.3 Meta-analysis results

#### 3.3.1 Body mass index (BMI) and waist-to-hip ratio (WHR)

**Figure 3:**
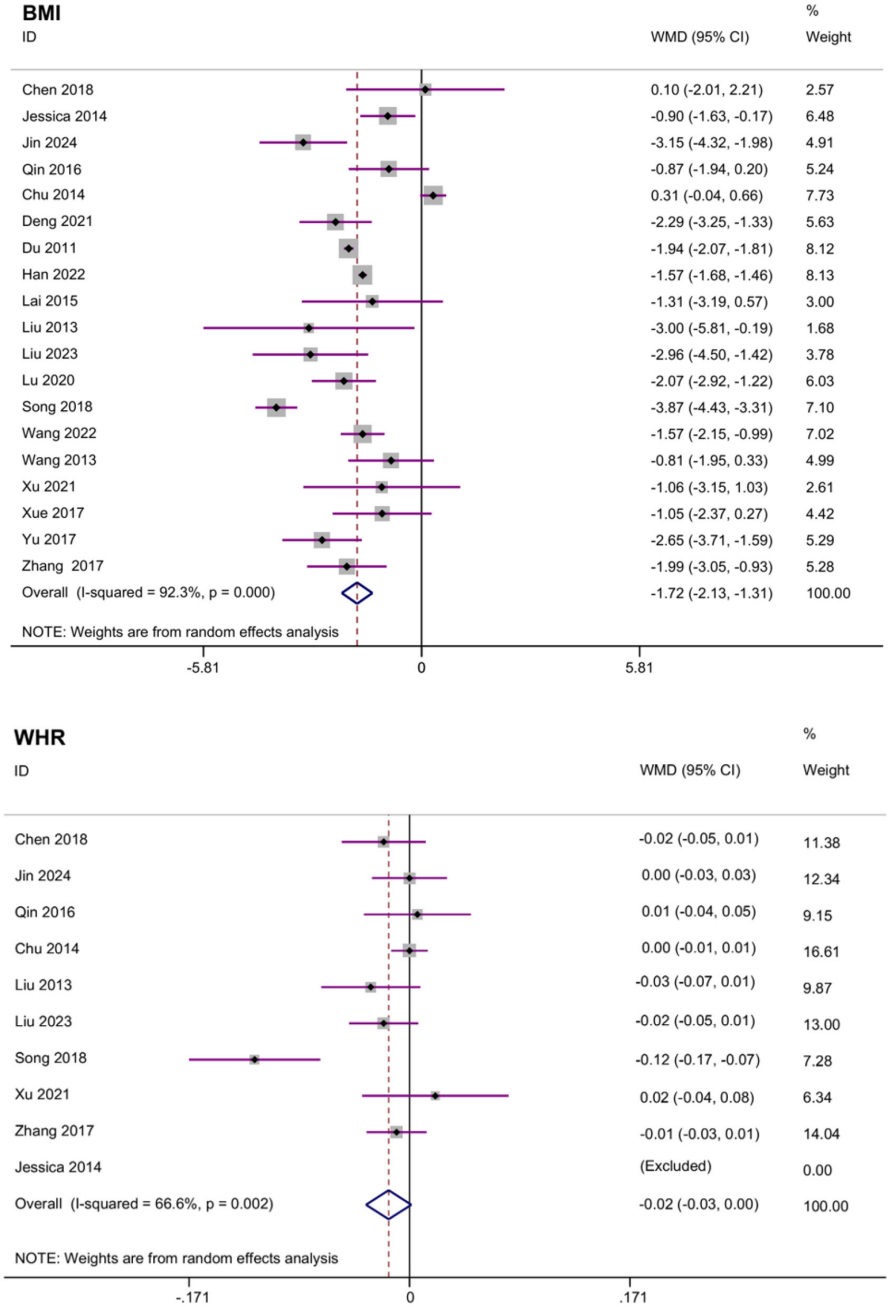
Meta analysis of BMI and WHR.

**Figure 4:**
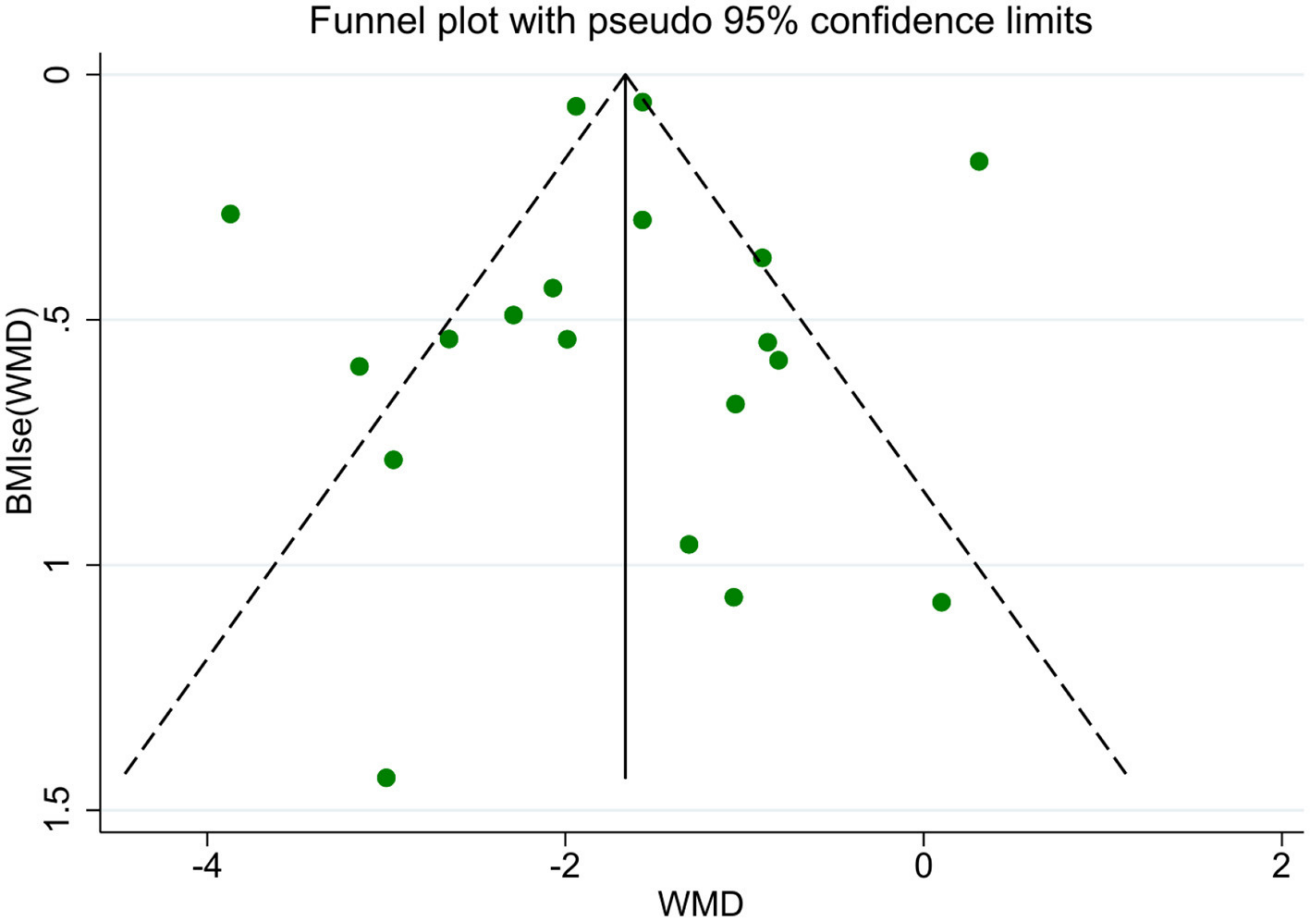
BMI funnel plot.

A total of 19 studies reported BMI results. The meta-analysis of BMI changes demonstrated a significant reduction following acupoint embedding therapy, with a WMD of −1.72 (95% CI: −2.13, −1.31; Figure 3), indicating a positive therapeutic effect. However, the analysis revealed high heterogeneity (*I*^2^ = 92.3%). Begg's Test (*P* = 0.944) and Egger's Test (*P* = 0.996) indicated no significant publication bias, and the funnel plot appeared relatively symmetrical (Figure 4). Sensitivity analysis confirmed that no single study had a substantial influence on the overall effect, ensuring the robustness of the results. A total of 10 studies reported WHR results. The meta-analysis for WHR showed significant effect of acupoint embedding therapy, with a WMD of −0.016 (95% CI: −0.034, 0.001; Figure 3), and moderate heterogeneity (*I*^2^ = 66.6%). Both Begg's Test (*P* = 0.602) and Egger's Test (*P* = 0.211) suggested no significant publication bias, with sensitivity analysis confirming the stability of the results (Figure 3).

#### 3.3.2 Clinical efficacy rate

**Figure 5:**
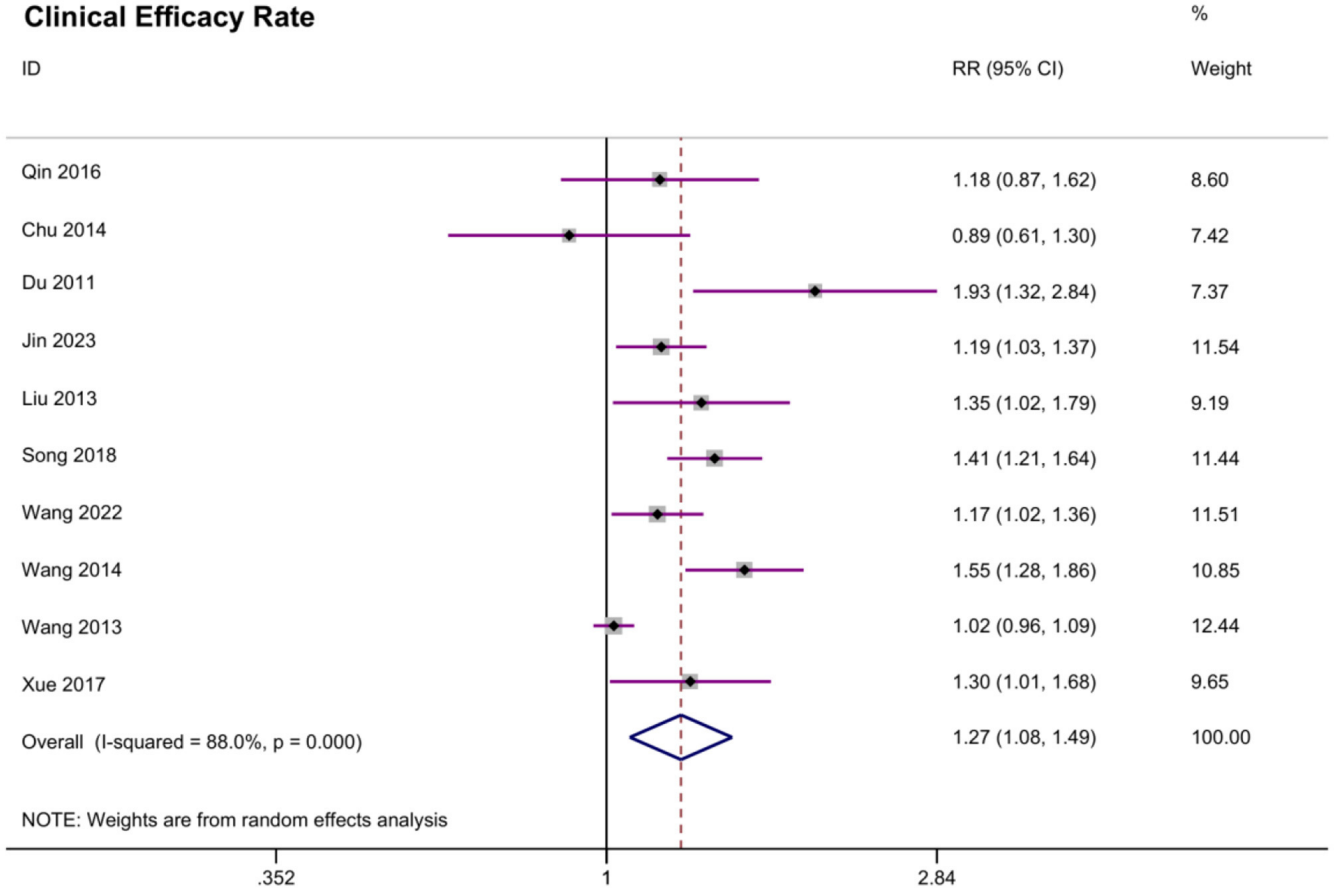
Meta analysis of clinical efficacy rate.

A total of 10 studies reported clinical efficacy rate. The meta-analysis yielded an overall significant effect of acupoint embedding therapy. The random effects model estimated the pooled RR at 1.265 (95% CI: 1.077, 1.487; Figure 5), suggesting a positive effect. The heterogeneity was moderate (*I*^2^ = 88.0%, *P* = 0.000), which indicates variability across the included studies. Begg's test (*P* = 0.529) did not reveal significant bias, and Egger's test indicated a potential bias (*P* = 0.039). The trim-and-fill analysis suggested potential publication bias, with five studies imputed on the left side of the funnel plot. The adjusted fixed-effect estimate increased from 1.159 (95% CI: 1.112–1.205) to 2.911 (2.792–3.035), while the random-effects estimate rose from 1.287 (1.136–1.438) to 2.942 (2.518–3.439). Significant heterogeneity persisted (*Q* = 151.000, *P* < 0.001), with between-study variance increasing from 0.046 to 0.079. These results indicate that the original analysis likely underestimated the true effect size due to missing studies. Despite this adjustment, the effect remained statistically significant, though the wider confidence intervals reflect greater uncertainty after accounting for potential publication bias. Sensitivity analysis indicated that no individual study had a substantial impact on the pooled estimate.

#### 3.3.3 Blood lipid indicators and HOMA-IR

A total of eight studies reported triglycerides (TG) results, seven studies reported total cholesterol (TC) results, three studies reported high density lipoprotein (HDL), and four studies reported low density lipoprotein (LDL) results. Three studies reported the HOMA-IR result. The meta-analysis for TG, TC, HDL, LDL, and HOMA-IR revealed no significant effects of acupoint embedding therapy on these outcomes. For TG, the WMD was −0.241 (95% CI: −0.43, −0.051; *P* = 0.013), and for TC, the WMD was −0.521 (95% CI: −0.912, −0.131; *P* = 0.009), both of which showed statistical significance. Similarly, the WMD for HDL was −0.024 (95% CI: −0.227, 0.18) (*P* = 0.821) and for LDL was −0.425 (95% CI: −1.006, 0.157; *P* = 0.152), both of which were also not significant. The meta-analysis of HOMA-IR indicated a reduction in insulin resistance with a WMD of −0.484 (95% CI: −1.111, 0.143; *P* = 0.13), but this also did not reach statistical significance. Heterogeneity across the studies was moderate to high for all indicators, with *I*^2^ values ranging from 56.9 to 84.8%. Publication bias was assessed using Begg's Test and Egger's Test, which showed no significant bias for any of the outcomes (*P* > 0.05). Sensitivity analysis confirmed the stability of the results, with no individual study significantly influencing the overall outcomes.

#### 3.3.4 Hormonal indicators

A total of five studies reported luteinizing hormone (LH) results, five studies reported follicle stumbling hormone (FSH) results, and two studies reported testosterone (T) results, three studies reported estradiol (E2). The meta-analysis for LH showed a moderate reduction following acupoint embedding therapy, with a WMD of −6.508 (95% CI: −9.772, −3.295; *P* < 0.001), and statistically significant effect was found. The heterogeneity was high (*I*^2^ = 95.8%), and Begg's Test (*P* = 0.806) and Egger's Test (*P* = 0.426) indicated there was no publication bias. Sensitivity analysis confirmed the robustness of the results. For FSH, a significant decrease was observed, with a WMD of −2.919 (95% CI: −4.963, −0.876; *P* = 0.005), and high heterogeneity (*I*^2^ = 97.8%). Begg's Test (*P* = 0.806) and Egger's Test (*P* = 0.064) showed no significant publication bias. Similarly, T showed no significant reduction, with a WMD of −0.647 (95% CI: −1.4, 0.105; *P* = 0.092). In contrast, for E2, the WMD was 7.131 (95% CI: 3.506, 10.756; *P* < 0.001), indicating a slight increase, and this was statistically significant. High heterogeneity (*I*^2^ = 88.3%) was present, and no publication bias was detected (Begg's Test *P* = 1.000; Egger's Test *P* = 0.19). Sensitivity analyses confirmed the stability of the findings for all hormones.

#### 3.3.5 Adverse events

The meta-analysis of adverse events included three studies with a total of 470 participants. The pooled risk ratio (RR) was 0.787 (95% CI: 0.554–1.116, *P* = 0.179), indicating no statistically significant difference in adverse events between the experimental and control groups. Heterogeneity was low (*I*^2^ = 0.0%, *P* = 0.423), supporting the use of a fixed-effects model. In Chen et al. (20), transient local pain, bruising, and itching occurred in both catgut embedding and sham groups. Qin et al. (21) reported decreased appetite (24/57) and mild implantation-site reactions (5/57), all resolving with conservative management. Song et al. (22) observed comparable low incidences of fever, bruising, and induration (11.0 vs. 10.0%, *P* = 0.947) between experimental and control groups.

#### 3.3.6 Subgroup analysis

In the subgroup analysis of main outcome BMI, we explored the sources of heterogeneity across the studies. In the original meta-analysis of BMI in nineteen studies, the heterogeneity was high, with an *I*^2^ value of 92.3%. However, when conducting the subgroup analysis, the sources of heterogeneity were explored, and a reduction in heterogeneity was observed across various subgroups. Specifically, for the sham embedding group, the *I*^2^ value decreased to 76.3%, indicating a moderate reduction in heterogeneity. The diet guidance group showed no heterogeneity (*I*^2^ = 0.0%). In the drug group, the heterogeneity decreased to 51.7%, reflecting a moderate reduction compared to the original analysis. On the other hand, the other acu-techniques group had a moderate *I*^2^ of 58.2% (Table 2).

**Table 2:** BMI subgroup analysis results.

**Subgroup**	**Number of studies (*n*)**	**95% CI**	***P* value**	***I* ^2^**
**Different control groups**				
Sham embedding group	4	−1.533 (−2.353, −0.712)	< 0.05	76.3%
Diet guidance	5	−1.936 (−2.058, −1.815)	< 0.05	0.0%
Drug group	4	−1.968 (−2.832, −1.104)	< 0.05	51.7%
Other acu-techniques	3	−0.246 (−1.188, 0.695)	0.608	58.2%
**Different intervention frequencies**				
Once every week	5	−0.785 (−1.96, 0.39)	0.19	86.4%
Once every 2 weeks	12	−2.178 (−2.555, −1.800)	< 0.05	87.7%
**Different treatment courses**				
6 weeks	3	−1.195 (−2.256, −0.135)	0.027	66.5%
8 weeks	3	−1.379 (−2.915, 0.157)	0.078	98.2%
12 weeks	9	−1.868 (−2.217, −1.519)	< 0.05	32.9%
**Different types of wires**				
Synthetic absorbable suture	5	−1.948 (−2.543, −1.352)	< 0.05	61.0%
Catgut	11	−1.606 (−2.425, −0.787)	< 0.05	95.3%
**Different age groups**				
< 30 years	7	−2.173 (−3.915, −0.431)	< 0.05	96.7%
30–50 years	6	−0.886 (−1.390, −0.383)	< 0.05	0%
>50 years	4	−1.932 (−2.054, −1.810)	< 0.05	0%

For the intervention frequency subgroups, the once every week group exhibited high heterogeneity (*I*^2^ = 86.4%), while the once every 2 weeks group had very high heterogeneity (*I*^2^ = 87.7%), indicating that different frequencies may not be the source of differences between studies. In terms of treatment duration, the heterogeneity in the 6-week group decreased to 66.5%, and in the 12-week group, it was reduced significantly to 32.9%. However, the 8-week group had very high heterogeneity (*I*^2^ = 98.2%). Finally, regarding wire types, the synthetic absorbable suture group had moderate heterogeneity (*I*^2^ = 61.0%), while the catgut group exhibited very high heterogeneity (*I*^2^ = 95.3%; Table 2).

Subgroup analysis by age revealed that the heterogeneity of intervention effects varied markedly across age groups. Participants under 30 years showed the largest BMI reduction but also exhibited substantial heterogeneity (*I*^2^ = 96.7%), indicating considerable variability in response within this group. In contrast, both the 30–50 years and >50 years groups demonstrated consistent effects with no observed heterogeneity (*I*^2^ = 0%), suggesting that age may be an important source of heterogeneity in treatment outcomes (Table 2).

## 4 Discussion

### 4.1 Main findings of the research

The most robust finding of our meta-analysis was the significant reduction in BMI among patients receiving acupoint embedding therapy. As shown in the BMI meta-analysis forest plot (Figure 3), the pooled weighted mean difference (WMD) in BMI was ~-1.72 (95% CI: −2.13 to −1.31), indicating a clinically meaningful weight reduction. This finding is consistent with results from previous studies on acupoint embedding for weight management (15, 17). However, high between-study heterogeneity was observed (*I*^2^ ≈ 92.3% for the BMI outcome, Figure 3), suggesting considerable variability in study populations, intervention protocols, or control treatments. The WHR reduction, though modest (WMD: −0.016), as depicted in Figure 3, further supports a potential role in central obesity management. Interestingly, the clinical efficacy rate was significant, but Egger's test (*P* = 0.039) and trim-and-fill analysis suggested possible publication bias, with an adjusted effect size increasing to RR = 2.942 (2.518–3.439). As Figure 5 illustrates, acupoint embedding continued to outperform the controls in terms of clinical efficacy even when potential publication bias was accounted for. This consistency reinforces the conclusion that the therapy's beneficial effect is genuine, despite the presence of some publication bias. Our meta-analysis demonstrated that acupoint embedding therapy produced a significant reduction in TG and TC, while changes in HDL, LDL and HOMA-IR did not reach statistical significance. These findings suggest that while acupoint embedding may favorably modulate overall lipid burden, its effects on lipoprotein subfractions and insulin resistance remain inconclusive. Early mechanism studies have shown that ACET may improve metabolic function by modulating key pathways (AMPK, PPAR-γ, mTOR) to enhance fat oxidation, glucose metabolism, and insulin sensitivity (23–25). Hormonal outcomes exhibited mixed effects: LH and FSH decreased significantly, while E2 increased. These findings align with prior evidence that acupoint stimulation may modulate hypothalamic-pituitary-gonadal axis activity, potentially benefiting conditions like polycystic ovary syndrome (PCOS) (26, 27). These therapies appear to regulate leptin activity and lipid profiles while promoting energy homeostasis through endocrine and circulatory effects (28, 29).

The pooled adverse event analysis showed no significant difference between acupoint embedding and control groups. Reported events (e.g., local pain, bruising, mild inflammation) were transient and manageable, supporting the therapy's safety. The comparable AE rates between active and sham procedures suggest that minor reactions may stem from needle insertion rather than the embedding material itself.

Subgroup analyses (Table 2) provided further insight into the sources of heterogeneity and the conditions under which acupoint embedding is most effective. As shown in Table 2, heterogeneity dropped to *I*^2^ = 0.0% among trials that provided uniform dietary guidance to all groups, indicating that standardizing co-interventions can markedly improve consistency across studies. Similarly, studies using synthetic absorbable sutures demonstrated lower heterogeneity (*I*^2^ = 61.0%), suggesting that embedding material may affect outcome stability. When compared to dietary guidance or medication alone, embedding therapy yielded a substantial and clinically meaningful reduction in BMI (mean difference: −1.936 and −1.968, respectively), with diet guidance as the control showing both strong effect size and no heterogeneity, underscoring the importance of standardized comparator arms. Further, analysis of intervention duration revealed that a 12-week treatment course was associated with the largest BMI reduction and relatively low heterogeneity (*I*^2^ = 32.9%), highlighting the benefit of extended therapy. Notably, the < 30 years group showed the greatest BMI reduction (−2.173), suggesting that younger women may be more responsive to embedding therapy. However, this subgroup had substantial heterogeneity (*I*^2^ = 96.7%), possibly reflecting variability in baseline metabolism, lifestyle, or adherence. In contrast, the >50 years group also achieved significant BMI reduction (−1.932) with complete consistency (*I*^2^ = 0%), indicating stable benefits among older women. The 30–50 years group had a smaller effect (−0.886) but similarly low heterogeneity. These trends may reflect that younger women benefit from higher metabolic rates (30), while older women may be more motivated or compliant (31), especially in the presence of comorbidities. Women aged 30–50 may experience more competing lifestyle pressures or metabolic plateaus, potentially limiting the intervention's impact (32).

In our included studies, acupoint selection for catgut embedding in obesity predominantly follows traditional acupuncture principles. Most protocols select 8–10 points per session, mainly targeting meridian points of the stomach, Ren Meridian, and Spleen Meridian. Commonly used acupoints are Tianshu (ST25), Zhongwan (CV12), Fenglong (ST40), Zusanli (ST36), and Guanyuan (CV4). Acupoint selection plays a crucial role in determining the therapeutic efficacy of ACET for women obesity. Studies show that more individualized or syndrome-based acupoint combinations can enhance outcomes. For instance, specialized abdominal point prescriptions has demonstrated superior effects on waist and abdominal circumference reduction compared to routine point selection (33, 34). Embedding at Jiaji (EX-B2) points, which are adjacent to spinal nerve branches, has shown greater improvements in BMI and body weight, likely through modulating visceral and autonomic functions (35).

### 4.2 Comparison with other systematic review and meta-analyses

Compared with other studies, Chen et al.'s (36) study performed a network meta-analysis of various acupuncture techniques targeting obesity with insulin resistance. Jiali et al. (37) directly compared ACET with manual acupuncture in simple obesity. Wujie et al. (15) combined acupuncture and embedding for abdominal obesity without isolating embedding effects; and Yue et al. (38) compared verum vs. sham ACET without examining hormonal changes. Our findings uniquely demonstrate that ACET not only reduces body weight and waist-hip ratio but also significantly alters luteinizing hormone, follicle-stimulating hormone, and estradiol levels, underscoring potential endocrine mechanisms in obesity management.

### 4.3 Limitations of the research

Despite the promising results, several limitations must be considered when interpreting these findings. One major limitation is the variation in study quality across the included research. Some studies (22, 39–46) showed concerns in areas such as randomization processes and incomplete reporting of outcomes, which may introduce bias and reduce the reliability of the conclusions. Additionally, there is substantial heterogeneity between the studies, particularly in terms of intervention protocols (e.g., catgut material, needle insertion depth, treatment frequency) and outcome measures (e.g., BMI, WC, metabolic markers). This variability makes it difficult to draw uniform conclusions about the optimal parameters for catgut embedding therapy.

### 4.4 Implications for future research

Future studies should standardize intervention protocols, including acupoint selection, and employ larger, multicenter, and longitudinal designs to strengthen statistical power and clarify long-term effects of catgut embedding for obesity. Mechanistic research should further explore how embedding at different sites influences neuroendocrine and metabolic pathways, focusing on hormones such as leptin, adiponectin, and insulin, as well as inflammatory and oxidative stress markers (47). Subgroup analysis suggest that personalized acupoint selection, tailored to age and metabolic status, may further optimize clinical outcomes. Refining protocols and integrating patient characteristics are essential for advancing the field.

## 5 Conclusion

Acupoint embedding therapy significantly reduced BMI and WHR and improved overall clinical efficacy in obese women, with additional benefits on triglycerides, total cholesterol and key reproductive hormones (LH, FSH, and E2). Effects on HDL, LDL, HOMA-IR, and testosterone were inconclusive. Treatment was well-tolerated, with adverse events comparable to controls. High heterogeneity, variable protocols and limited sample sizes warrant larger, standardized trials with longer follow-up to confirm these findings and elucidate underlying mechanisms.

## Data Availability

The original contributions presented in the study are included in the article/Supplementary material, further inquiries can be directed to the corresponding authors.
