# Supplementary material for: The effects of acupoint catgut embedding therapy on anthropometric parameters and endocrine function in obese women: a systematic review and meta-analysis
Source: Front Nutr. 2025 Jul 1;12:1583556. doi: 10.3389/fnut.2025.1583556 (PMC12259452; doi:10.3389/fnut.2025.1583556)
Supplement: Supplementary file 1 [file Data_Sheet_1.PDF]

An official website of the United States government
Here's how you know

PubMed Advanced Search Builder

PubMed®

Add terms to the query box

All Fields

Enter a search term

ADD

Show Index

Query box

Enter / edit your search query here

Search

History and Search Details

Search	Actions	Details	Query	Results	Time
#4	...		Search: **((((Female or Women or Women's[MeSH Terms])) OR (Female[Title/Abstract] OR Women[Title/Abstract] OR Women's[Title/Abstract])) AND ((catgut implantation at acupoint or catgut embedding or Catgut-implantation or catgut embedding or catgut-point embedding[MeSH Terms]) OR (catgut implantation at acupoint[Title/Abstract] OR catgut embedding[Title/Abstract] OR Catgut-implantation[Title/Abstract] OR catgut embedding[Title/Abstract] OR catgut-point embedding[Title/Abstract]))) AND ((Obesity or Adiposity or Body Weight or Obesity, Abdominal or Abdominal Obesities or Obesities, Abdominal or Abdominal Obesity or Central Obesity or Central Obesities or Obesities, Central or Obesity, Central or Obesity, Visceral or Visceral Obesity or Obesities, Visceral or Visceral Obesities[MeSH Terms]) OR (Obesity[Title/Abstract] OR Adiposity[Title/Abstract] OR Body Weight[Title/Abstract] OR Obesity, Abdominal[Title/Abstract] OR Abdominal Obesities[Title/Abstract] OR Obesities, Abdominal[Title/Abstract] OR Abdominal Obesity[Title/Abstract] OR Central Obesity[Title/Abstract] OR Central Obesities[Title/Abstract] OR Obesities, Central[Title/Abstract] OR Obesity, Central[Title/Abstract] OR Obesity, Visceral[Title/Abstract] OR Visceral Obesity[Title/Abstract] OR Obesities, Visceral[Title/Abstract] OR Visceral Obesities[Title/Abstract]))**	29	03:27:22
#3	...		Search: **(Obesity or Adiposity or Body Weight or Obesity, Abdominal or Abdominal Obesities or Obesities, Abdominal or Abdominal Obesity or Central Obesity or Central Obesities or Obesities, Central or Obesity, Central or Obesity, Visceral or Visceral Obesity or Obesities, Visceral or Visceral Obesities[MeSH Terms]) OR (Obesity[Title/Abstract] OR Adiposity[Title/Abstract] OR Body Weight[Title/Abstract] OR Obesity, Abdominal[Title/Abstract] OR Abdominal Obesities[Title/Abstract] OR Obesities, Abdominal[Title/Abstract] OR Abdominal Obesity[Title/Abstract] OR Central Obesity[Title/Abstract] OR Central Obesities[Title/Abstract]**	1,080,868	03:25:10

Search	Actions	Details	Query	Results	Time
			**OR Obesities, Central[Title/Abstract] OR Obesity, Central[Title/Abstract] OR Obesity, Visceral[Title/Abstract] OR Visceral Obesity[Title/Abstract] OR Obesities, Visceral[Title/Abstract] OR Visceral Obesities[Title/Abstract])**		
#2	•••		Search: **(catgut implantation at acupoint or catgut embedding or Catgut-implantation or catgut embedding or catgut-point embedding[MeSH Terms]) OR (catgut implantation at acupoint[Title/Abstract] OR catgut embedding[Title/Abstract] OR Catgut-implantation[Title/Abstract] OR catgut embedding[Title/Abstract] OR catgut-point embedding[Title/Abstract])**	332	03:24:31
#1	•••		Search: **((Female or Women or Women's[MeSH Terms])) OR (Female[Title/Abstract] OR Women[Title/Abstract] OR Women's[Title/Abstract])**	10,716,920	03:23:18

Showing 1 to 4 of 4 entries

FOLLOW NCBI

Connect with NLM

National Library of Medicine
8600 Rockville Pike
Bethesda, MD 20894

Web Policies
FOIA
HHS Vulnerability Disclosure

Help
Accessibility
Careers

NLM NIH HHS USA.gov

Cochrane Library

Access provided by: **China Medical University**

我们注意到您的浏览器语言为简体中文
您可以在任何页面的顶部选择倾向的语种，阅读翻译成该语种的Cochrane系统综述内容

更改为简体中文

Advanced Search

Search manager

Save this search | View/Share saved searches | ? Search help

Print search history

#1	(Female or Women or Women's):ti,ab,kw AND (catgut implantation at acupoint or catgut embedding or Catgut-implantation or catgut embedding or catgut-point embedding):ti,ab,kw AND (Obesity or Adiposity or Body Weight or Obesity, Abdominal or Abdominal Obesities or Obesities, Abdominal or Abdominal Obesity or Central Obesity or Central Obesities or Obesities, Central or Obesity, Central or Obesity, Visceral or Visceral Obesity	S	Limits	44
	(Word variations have been searched)			
#2	Type a search term or use the S or MeSH	S / MeSH	Limits	N/A

Clear all

Highlight orphan lines

Save this search | View/Share saved searches | ? Search help

Print search history

Filter your results

Cochrane Reviews 0 | Cochrane Protocols 0 | **Trials 44** | Editorials 0 | Special Collections 0 | More

Results

#1 AND #2 AND #3

Search > Mapping Date Sources Fields Quick limits EBM Pub. types

Collapse

#4	#1 AND #2 AND #3	56
#3	**obesity**:ti,ab,kw OR **adiposity**:ti,ab,kw OR **'body weight'**:ti,ab,kw OR **'obesity, abdominal'**:ti,ab,kw OR **'abdominal obesities'**:ti,ab,kw OR **'obesities, abdominal'**:ti,ab,kw OR **'abdominal obesity'**:ti,ab,kw OR **'central obesity'**:ti,ab,kw OR **'central obesities'**:ti,ab,kw OR **'obesities, central'**:ti,ab,kw OR **'obesity, central'**:ti,ab,kw OR **'obesity, visceral'**:ti,ab,kw OR **'visceral obesity'**:ti,ab,kw OR **'obesities, visceral'**:ti,ab,kw OR **'visceral obesities'**:ti,ab,kw OR **'obesity'**/exp OR	1,707,392

Results Filters Apply >

- Sources
- Drugs
- Diseases
- Devices
- Floating Subheadings
- Age
- Gender
- Study types
- Publication types
- Journal titles
- Publication years
- Authors

Drug Manufacturers

Device Trade Names

Device Manufacturers

Apply >

abdominal' OR **'abdominal obesity'**/exp OR **'central obesity'**/exp OR **'central obesities'** OR **'obesities, central'** OR **'obesity, central'** OR **'obesity, visceral'** OR **'visceral obesity'**/exp OR **'obesities, visceral'** OR **'visceral obesities'**

#2 '(Edit Email alert RSS feed

acupoint':ti,ab,kw OR **'catgut implantation'**:ti,ab,kw OR **'catgut embedding'**:ti,ab,kw OR **'catgut-point embedding'**:ti,ab,kw OR **'catgut implantation at acupoint'** OR **'catgut implantation'**/exp OR **'catgut embedding'**/exp OR **'catgut-point embedding'**

#1 **female**:ti,ab,kw OR **women**:ti,ab,kw OR **womens**:ti,ab,kw OR **'female'**/exp OR **'women'**/exp OR **womens** 13,535,481

56 results for search #4 Set email alert Set RSS feed Search details

Index miner

1 — 25

56 Selected: 56 (clear)Sł

简体中文 产品

Web of Science™ 检索 登录 注册

< 返回基本检索

高级检索式生成器

文献 研究人员

选择数据库: **Web of Science 核心合集** 引文索引: **All**

将检索词添加到检索式预览

所有字段 | 示例: liver disease india singh | AND | 添加到检

更多选项 检索帮助

检索式预览

TS=(Obesity or Adiposity or Body Weight or Obesity, Abdominal or Abdominal Obesities or Obesities, Abdominal or Abdominal Obesity or Central Obesity or Central Obesities or Obesities, Central or Obesity, Central or Obesity, Visceral or Visceral Obesity or Obesities, Visceral or Visceral Obesities)

+ 添加日期范围 × 清除 添加到历史

布尔运算符 : AND, OR, NOT

字段标识 : 排序方式 Default

- TS=主题
- TI=标题
- AB=摘要
- AU=[作者]
- AI=作者标识符
- AK=作者词

- GP=[团体作者]
- ED=编者
- KP=Keyword Plus®
- SO=[出版物标题]
- DO=DOI
- PY=出版年
- CF=会议
- AD=地址
- OG=[所属机构]
- OO=组织
- SG=下属组织
- SA=街道地址
- CI=城市
- PS=省/州
- CU=国家/地区
- ZP=邮编 (邮政编码)
- FO=基金资助机构
- FG=授权号
- FD=基金资助详情
- FT=基金资助信息
- SU=研究方向
- WC=Web of Science 类别
- IS= ISSN/ISBN
- UT=入藏号
- PMID=PubMed ID
- DOP=出[illegible]期
- LD=索[illegible]
- PUBL=[illegible]
- ALL=所[illegible]
- FPY=最[illegible]年
- EAY=在[illegible]年份
- SDG=可[illegible]发展目[illegible]
- TMAC=[illegible]别引文[illegible]
- TMSO=[illegible]别引文[illegible]
- TMIC=[illegible]别引文[illegible]

会话检索式

根据您在此会话中的检索构建新检索式。

0/4 组配检索式 导出 清除历[illegible]

4	#3 AND #2 AND #1	16	添加到检索式
3	TS=(Obesity or Adiposity or Body Weight or Obesity, Abdominal or Abdominal Obesities or Obesities, Abdominal or Abdominal Obesity or Central Obesity or Central Obesities or Obesities, Central or Obesity, Central or Obesity, Visceral or Visceral Obesity or Obesities, Visceral or Visceral Obesities)	921,100	添加到检索式

☐	2	TS=(catgut implantation at acupoint or catgut embedding or Catgut-implantation or catgut embedding or catgut-point embedding)	225
☐	1	Female or Women or Women's (主题)	3,375,310

添加到检索式

添加到检索式

University of Manchester

MIMAS Web of Knowledge Home Page -

Clarivate

Accelerating innovation

© 2025 Clarivate

培训门户

产品支持

数据修正

隐私声明

新闻通讯

版权声明

Cookie 政策

使用条款

管理 cookie 首选项

京ICP备20012568号 京公网安备11010802032525号

CBPDT 确认

关注我们

菜单

Scopus

欢迎使用更直观、更高效的搜索体验。 查看新功能

保存检索

设置检索通知

高级查询

检索范围
论文标题、摘要、关键词

关键字检索 *
female OR women OR women's

AND

检索范围
论文标题、摘要、关键词

关键字检索
catgut AND implantation AND at AND acupoint OR catgut AND embedding OR catgut-implantation OR cat

AND

检索范围
论文标题、摘要、关键词

关键字检索
obesity OR adiposity OR body AND weight OR abdominal AND obesities OR abdominal AND obesity OR cen

+ 添加检索字段

重置 检索

文献 预印本 测试版 专利 辅助文献 研究数据

您是否要检索: (TITLE-ABS-KEY (female OR women OR women's) AND TITLE-ABS-KEY (catgut AND implantation AND **rat** AND acupoint OR catgut AND embedding OR catgut-implantation OR catgut AND embedding OR catgut-point AND embedding) AND TITLE-ABS-KEY (Obesity or Adiposity or Body Weight or Abdominal Obesities or Abdominal Obesity or Central Obesity or Central Obesities or Visceral Obesity or Visceral Obesities))

找到 3 篇文献

分析结果

细化搜索

在搜索结果内搜索

筛选器

年份

范围 单个

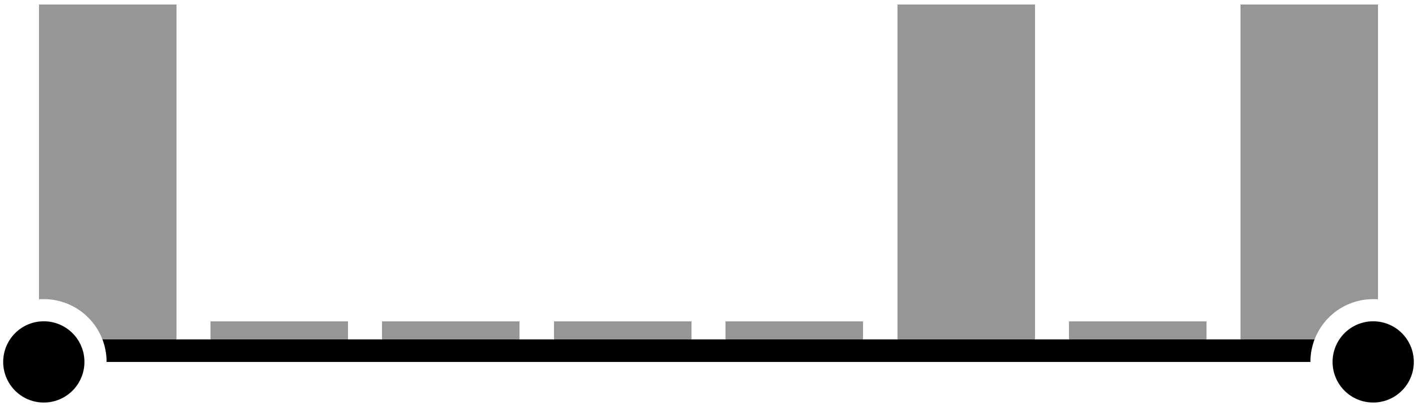

从 – 到

Help

学科类别

小助理

Medicine 2
您好，请问您想了解什么？
Biochemistry, Genetics and Molecular Biology 1

小助理可能会犯错，请核查重要信息

概括标题摘要 提取关键词 解释关键词 推荐相关论文 推荐相关论文（未读的） 更换关键词 显示更多关键词 翻译摘要 调整小助理位置 解答更多问题

文献类型

关闭小助理

Article 3

语言

English 3
Spanish 1

关键字

Article 3
Catgut 3
Catgut Embedding 3
Controlled Study 3
Female 3

显示全部

国家/地区

来源出版物类型

来源出版物名称

作者姓名

出版阶段

归属机构

资金赞助商

开放获取

导出筛选器数量

全部 导出 下载 引文概览 ••• 更多 显示所有摘要 排序依据 日期（最近）

	文献标题	作者	来源出版物	年份	引文
1	Article<br>**Laser beam application versus catgut implantation in obese diabetic patients | Aplicación de rayo láser frente a implantación de catgut en pacientes diabéticos con obesidad**<br>查看摘要 get it BIT View at Publisher 相关文献	Becerril Chavéz, F., Carlín Vargas, G., Freyre Valenzuela, A.S., Aragón García, M.	Revista Internacional de Acupuntura, 16(3), 100187	2022	0
2	Article • *开放获取*<br>**Evidence for acupoint catgut embedding treatment and TRPV1 gene deletion increasing weight control in murine model**<br>SCI升级版 医学2区 SCI基础版 医学3区 IF 5.7<br>查看摘要 get it BIT View at Publisher 相关文献	Inprasit, C., Huang, Y.-C., Lin, Y.-W.	International Journal of Molecular Medicine, 45(3), 页 779–792	2020	13
3	Article<br>**Clinical efficacy of simple obesity treated by catgut implantation at acupoints**<br>SCI升级版 医学4区 SCI基础版 医学3区 IF 2.2 SWJTU A	Yan, R.-H., Liu, X.-M., Bai, J., ... Yu, J., Gu, J.-S.	Chinese Journal of Integrative Medicine, 21(8), 页 594–600	2015	8

Help

小助理

(主题：埋线 + 注线 + 穴位埋线）AND（主题：腹型肥胖 + 肥胖 + 腹...

（主题：埋线 + 注线 + 穴位埋线）AND（主题：腹型肥胖 + 肥胖 + 腹部肥胖）AND（主题：女性 + 女）

文献检索

(U=女性 OR U=女) AND (U=肥胖 OR U=腹部肥胖 OR U=腹型肥胖) AND (U=埋线 OR U=穴位埋线 OR U=注线)

全部	中文	外文	期刊论文	学位论文	会议论文	专利	标准
133	125	8	69	51	13	0	0

二次检索

主题 请输入检索词

在结果中检索 在结果中去除

获取类型

有全文

免费开放

年份

近3年 近5年 近10年 重置

2024 (6)

2023 (8)

共 69 条结果， 以下是1 - 20条

全选 已选:20 清除 批量下载 批量引用 批量综述 AI

1. B超下不同层次穴位埋线对女性腹型肥胖及排便功能的近期疗效及安全性观察

【期刊论文】 • 韩桂华 1,2 倪光夏 1 孙建华 3 裴丽霞 3 +6位作者 • 《针刺研究》 PubMed CSCD 北大[illegible] [illegible]A

机构: [1] 南京中医药大学针灸推拿学院 [2] 南京市鼓楼区凤凰社区卫生服务中心针灸科 [3] 南京中医药大学附属医院针灸康复科

摘要: 目的:探讨B超下不同层次穴位埋线对女性腹型肥胖及排便功能的近期疗效和安全性。方法:将102例女性腹型肥胖患者随机分为深埋[illegible]落1例组将线体埋置在脂肪层+肌肉层,浅埋线组将线体埋置在脂肪层,假埋线组无线体埋置。埋线穴位为中脘、关元、关门(双)、天枢(双)、带脉(双[illegible]每2周

关键词: 腹型肥胖； 随机对照试验； B超； 排便功能； 穴位埋线

万方数据 WANFANG DATA 知识服务平台　学习中心　应用　会员　清华大学　登录 / 注册　简　繁

高级检索　**专业检索**　作者发文检索　自然语言检索　了解专业检索　推荐检索词

文献类型： 全部　期刊论文　学位论文　会议论文　专利　中外标准　科技成果　法律法规　科技报告　地方志

通用　全部　主题　题名或关键词　题名　第一作者　作者单位　作者　关键词　摘要　DOI

逻辑关系　and(与)　or(或)　not(非)

(主题:("埋线") or 主题:("穴位埋线") or 主题:("注线")) and (主题:("腹部肥胖") or 主题:("腹型肥胖") or 主题:("肥胖")) and (主题:("女性") or 主题:("女"))

113/800

出版时间：不限 - 至今　智能检索：中英文扩展　主题词扩展

检索　**检索历史**

温馨提示

1. 选择资源类型后，可使用对应资源的独有字段检索。
2. 运算符含义：
   - AND：逻辑与，所有词同时出现在文献中。
   - OR：逻辑或，至少一个词出现在文献中。
   - NOT：逻辑非，后面的词不出现在文献中。
   - " "：精确匹配，引号内容作为整体进行检索。
   - ( )：限定检索顺序，括号内容作为一个子查询。
3. 逻辑运算优先级顺序：
   ( ) > NOT > AND > OR。
4. 运算符建议使用英文半角输入形式。

主题:("埋线") or 主题:..　主题:("腹部肥胖") or ...　主题:("女性") or 主题:..　(主题:("埋线") or 主题.

检索表达式： (主题:("埋线") or 主题:("穴位埋线") or 主题:("注线")) and (主题:("腹部肥胖") or 主题:("腹型肥胖") or 主题:("肥胖")) and (主题:("女性") or 主题:("女"))

找到 **70** 条文献

获取范围

已选择 70 条　清除　批量引用　批量下载　**排序：** 相关度　出版时间　被引频次　下载量　显示 50 条

个人文献检测入口　万方检测京东店铺　手机版　联系客服

("女性"[常用字段:智能] OR "女"[常用字段:智能]) AND ("腹部肥胖"[常用字段:智能] OR "肥胖 检索 二次检索

结果筛选

来源

中文文献(67)

西文文献(0)

博硕论文(0)

科普文献(8)

主题 +

学科 +

时间 +

期刊 +

作者 +

机构 +

基金 +

地区 +

文献类型 +

期刊类型 +

检索条件 : ("女性"[常用字段:智能] OR "女"[常用字段:智能]) AND ("腹部肥胖"[常用字段:智能] OR "肥胖"[常用字段:智能] OR "腹型
[常用字段:智能] OR "腹性肥胖"[常用字段:智能]) AND ("埋线"[常用字段:智能] OR "穴位埋线"[常用字段:智能] OR "注线"[常用字段

年代 - 检索历史

详细检索表达式

(("女性"[常用字段] OR "Femininity"[常用字段] OR "女性"[主题词]) OR "女"[常用字段]) AND (("腹部肥胖"[常用字段] OR "Abdominal Obesity"[常用字段] OR "中心型肥胖"[常用字段] OR "向心性肥胖"[常用字段] OR "内脏肥胖"[常用字段] OR "内脏性肥胖"[常用字段] OR "肥胖, 腹部"

检索

您好，请问您想了解什么？ 文献标题 作者 来源出版物 年份 引文

查看摘要 get it BIT View at Publisher 相关文献 小助手可能会犯错，请核查重要信息

概括标题+摘要 提取关键词 解释关键词 推荐相关论文 推荐相关论文（未读的） 更换关键词 显示更多关键词 翻译摘要 调整小助理位置 解答更多问题

关闭小助理 Scopus AI New 利用近期工作概要查找研究参考文献。 试用 Scopus AI

显示 10 结果

返回页首

Help

小助理

您好，请问您想了解什么？
小助理可能会犯错，请核查重要信息
概括标题+摘要 提取关键词 解释关键词 推荐相关论文 推荐相关论文（未读的） 更换关键词 显示更多关键词 翻译摘要 调整小助理位置 解答更多问题
关闭小助理

关于 Scopus

语言

客户服务

ELSEVIER
条款与条件 ↗ 隐私策略 ↗
All content on this site: Copyright © 2025 Elsevier B.V. ↗, its licensors, and contributors. All rights are reserved, including those for text and data mining, AI training, and similar technologies. For all open access content, the relevant licensing terms apply.
我们使用 Cookie 来帮助提供和增强我们的服务并量身定制显示的内容。继续即表示您同意使用 Cookie ↗。
RELX™
Help
小助理
